# Improving Safe Oxygen Therapy Through Education: A Quality Improvement Project

**DOI:** 10.7759/cureus.117514

**Published:** 2026-10-05

**Authors:** Naazira Begum, K Ferdose Uzma, Tooba Fatima Iram, Haroon Abdullah, Shaheed Antlee, Suraiya Begum

**Affiliations:** 1 Emergency Medicine, Mythri Hospitals, Hyderabad, IND; 2 Medicine, Kaloji Narayana Rao University of Health Sciences, Hyderabad, IND; 3 Cardiac Critical Care, Care Hospitals, Hyderabad, IND; 4 Dermatology, King Salman Bin Abdulaziz Medical City, Madinah, SAU; 5 Pathology, King Salman Bin Abdulaziz Medical City, Madinah, SAU

**Keywords:** emergency department, oxygen stewardship, oxygen weaning, patient safety, quality improvement

## Abstract

Introduction: Oxygen therapy is a commonly used intervention in the emergency department (ED) and can be lifesaving when appropriately indicated. However, inappropriate administration, inadequate reassessment, and delayed weaning may result in unnecessary oxygen exposure and potential harm. This quality improvement project aimed to improve nurses’ knowledge and awareness of safe oxygen therapy practices through targeted education and a structured oxygen review prompt incorporated into routine clinical handover.

Methods: A two-cycle Plan-Do-Study-Act (PDSA) methodology was undertaken in the ED of a tertiary care hospital in India. Thirty ED nurses participated voluntarily and completed a structured 10-item questionnaire assessing knowledge and awareness of safe oxygen therapy, including oxygen as a medication, target oxygen saturation ranges, monitoring, potential harms of excessive oxygen administration, and oxygen review, reassessment, and weaning principles. The first PDSA cycle consisted of alternate-day educational sessions delivered over two weeks. The second cycle incorporated a structured oxygen review prompt into routine nursing and medical handovers for a further two weeks.

Results: At baseline, 18/30 nurses (60%) achieved satisfactory questionnaire performance. This increased to 22/30 (73.3%) following PDSA cycle 1 and to 28/30 (93.3%) following PDSA cycle 2. The observed increase represented an improvement in overall questionnaire-based knowledge and awareness across the three assessment stages. However, actual bedside oxygen prescribing, reassessment, and weaning practices were not directly observed or audited.

Conclusion: The project identified important gaps in nurses’ knowledge of target oxygen saturation ranges and highlighted the importance of reinforcing safe oxygen review and reassessment. Sequential implementation of targeted education and a structured oxygen review prompt incorporated into routine clinical handover was associated with improved questionnaire performance among participating ED nurses. Embedding safe oxygen principles and oxygen reassessment into existing clinical workflows may provide a practical approach to supporting staff knowledge and promoting safer, more individualized oxygen therapy in the ED. Further evaluation using objective clinical measures and longer-term follow-up is needed to determine whether improvements in questionnaire performance translate into sustained changes in actual oxygen therapy practice.

## Introduction

Oxygen therapy is one of the most commonly used treatments in the emergency department (ED) and is an essential intervention for patients with hypoxemia and respiratory distress [1]. Although oxygen can be lifesaving when clinically indicated, inappropriate or prolonged oxygen therapy may expose patients to unnecessary treatment and potential harm. Therefore, oxygen should be treated as a medication, with appropriate prescribing, monitoring, regular reassessment, and adjustment according to the patient’s clinical condition.

Current clinical practice recommends prescribing oxygen to achieve an appropriate target oxygen saturation range based on the individual patient’s clinical condition. However, as a patient’s condition improves, oxygen therapy may not always be reviewed or reduced in a timely manner. This can result in patients continuing to receive supplemental oxygen despite no longer requiring it. This is particularly important in patients with chronic obstructive pulmonary disease (COPD) who are at risk of hypercapnic respiratory failure, in whom excessive oxygen administration can worsen hypercapnia and lead to respiratory acidosis. Appropriate oxygen prescribing, regular reassessment, and timely weaning are therefore essential to ensure that patients receive oxygen within their recommended target saturation range.

The ED presents particular challenges to consistent oxygen reassessment due to high patient turnover, competing clinical priorities, and heavy workloads. These factors may contribute to missed opportunities to review oxygen requirements and appropriately wean or discontinue therapy. Unnecessary or prolonged oxygen administration may expose patients to hyperoxemia and, in some circumstances, oxygen-related toxicity. Hyperoxemia has also been associated with an increased risk of adverse outcomes, including mortality, potentially through oxidative stress and free-radical-mediated tissue injury. Improving awareness of appropriate oxygen prescribing and encouraging regular reassessment may therefore help reduce unnecessary oxygen exposure and promote safer, more individualized patient care [2].

This quality improvement project was undertaken to assess nurses’ knowledge and awareness of safe oxygen therapy practices in the ED and to identify areas for improvement. The project used targeted education and a structured oxygen review prompt integrated into routine clinical handover to reinforce key principles of oxygen reassessment, target saturation, and appropriate oxygen reduction or discontinuation.

## Materials and methods

This quality improvement project was conducted in the ED of a tertiary care hospital in India, where oxygen therapy is frequently initiated for patients presenting with a wide range of acute medical and surgical conditions. The objective of this quality improvement project was to improve nurses’ knowledge and awareness of safe oxygen therapy practices in the ED. The ED was selected as the setting because of the high volume and rapid turnover of patients, frequent initiation and adjustment of supplemental oxygen, and the need for timely reassessment of oxygen requirements as patients’ clinical conditions change.

A two-cycle Plan-Do-Study-Act quality improvement project was conducted among 30 ED nurses. Assessments were performed at baseline, following PDSA cycle 1, and following PDSA cycle 2 using a structured 10-item questionnaire evaluating knowledge and awareness of safe oxygen therapy practices. The primary outcome was the proportion of participating ED nurses achieving satisfactory performance on the 10-item oxygen therapy questionnaire at each assessment stage. Participation was voluntary, and informed consent was obtained from all participants.

Baseline assessment

A baseline assessment was undertaken before implementing the quality improvement interventions. A structured questionnaire was administered to 30 participating nurses to assess their knowledge and awareness of safe oxygen therapy practices. The questionnaire was developed specifically for this project by the project team based on established principles of safe oxygen therapy and relevant clinical guidelines. It covered key domains, including oxygen prescription, target oxygen saturation ranges, monitoring, potential harms of excessive oxygen administration, reassessment, and oxygen reduction, weaning, and discontinuation.

The questionnaire was reviewed internally for relevance and clarity before distribution. However, formal validation and structured pilot testing were not undertaken, which is acknowledged as a limitation of the project.

Each question had a predefined correct answer based on the principles of safe oxygen therapy, with one point assigned for each correct response. The questionnaire consisted of 10 questions, and the number of nurses who answered all questions correctly was recorded at each assessment stage and expressed as a proportion and percentage of the 30 participants. Satisfactory performance was predefined as answering all 10 questionnaire items correctly (10/10; 100%). Each correct response was awarded one point, giving a total possible score of 10. The proportion of nurses achieving a score of 10/10 was compared across the baseline assessment, following PDSA cycle 1, and following PDSA cycle 2.

At baseline, 18 of 30 nurses answered all questions correctly. Following the first PDSA cycle and the associated teaching sessions, the same questionnaire was administered, with 22 of 30 nurses answering all questions correctly. Following the second PDSA cycle, which incorporated the structured handover prompt, this increased further to 28 of 30 nurses. This approach was used to assess changes in overall questionnaire performance across the three assessment stages.

Particular emphasis was placed on administering oxygen to achieve an appropriate target saturation range rather than continuing oxygen automatically after initial treatment. The questionnaire also assessed awareness of the need to reassess oxygen requirements as patients’ clinical conditions improved and the importance of cautious oxygen administration in patients at risk of hypercapnic respiratory failure.

PDSA cycle 1: education and training

The first PDSA cycle focused on improving nurses’ knowledge and awareness through structured education and training. The baseline questionnaire findings were reviewed to identify areas requiring particular attention, including oxygen as a medication, target oxygen saturation ranges, the potential adverse effects of excessive oxygen administration, and the importance of regular reassessment and appropriate oxygen weaning.

Alternate-day teaching sessions were conducted over a two-week period for ED nurses. The sessions were arranged to minimize disruption to clinical activities and to facilitate participation among nurses working different shifts.

The educational intervention addressed the principles of safe oxygen therapy, including appropriate indications for oxygen administration, prescribing oxygen to a defined target saturation range, monitoring oxygen saturation, and recognizing when supplemental oxygen may no longer be clinically required. The sessions also discussed the potential consequences of inappropriate or excessive oxygen administration, including hyperoxemia and the risk of worsening hypercapnia in susceptible patients, such as those with COPD.

Practical oxygen reassessment was incorporated into the teaching. Nurses were encouraged to review the patient’s current oxygen saturation, prescribed target saturation range, clinical indication for oxygen therapy, oxygen delivery device, flow rate or concentration, and whether oxygen reduction or discontinuation should be considered. Common clinical scenarios encountered in the ED were discussed to reinforce the application of these principles in routine practice. These observations informed the development of the second PDSA cycle.

PDSA cycle 2: reinforcement of oxygen review

The second PDSA cycle aimed to reinforce the knowledge gained during the educational intervention and facilitate its translation into routine clinical practice. A structured oxygen review prompt was incorporated into routine clinical handover for a further two-week period.

The prompt provided a systematic framework for reviewing patients receiving supplemental oxygen. Nurses were encouraged to consider the patient’s current oxygen requirement, oxygen saturation, prescribed target saturation range, clinical indication for continued oxygen therapy, oxygen delivery device, and current flow rate or concentration.

The handover review also prompted consideration of whether the patient’s clinical condition had improved sufficiently to allow reduction of oxygen and whether supplemental oxygen could be safely discontinued. Nurses were encouraged to communicate changes in oxygen requirements during handover and to escalate concerns to the responsible clinician when oxygen requirements increased, target saturation was not achieved, or the indication for ongoing oxygen therapy was unclear.

Embedding oxygen review within routine clinical handover was intended to incorporate reassessment into an existing workflow rather than creating an additional standalone task. This was considered particularly relevant in the ED, where rapid patient turnover and competing clinical priorities can contribute to continuation of oxygen therapy without timely reassessment.

Assessment and evaluation of impact

Assessments were conducted at baseline, following PDSA cycle 1, and following PDSA cycle 2. The structured questionnaire was administered to the same 30 nurses at each assessment stage. The questionnaire retained the same core knowledge domains. The proportion of nurses achieving satisfactory performance, defined as answering all 10 questionnaire items correctly, was compared across the three assessment stages.

The evaluation focused on changes in understanding of oxygen as a medication, recommended target oxygen saturation ranges, oxygen saturation monitoring, potential harms associated with excessive oxygen administration, the importance of regular oxygen reassessment, and appropriate oxygen reduction or discontinuation.

The first PDSA cycle was primarily educational, while the second cycle focused on reinforcing the educational messages through integration of a structured oxygen review prompt into routine clinical handover (Figure 1). The combined evaluation was therefore intended to determine whether education supported by a simple workflow-based reminder was associated with improved awareness of safe oxygen therapy practices among participating nurses.

**Figure 1:**
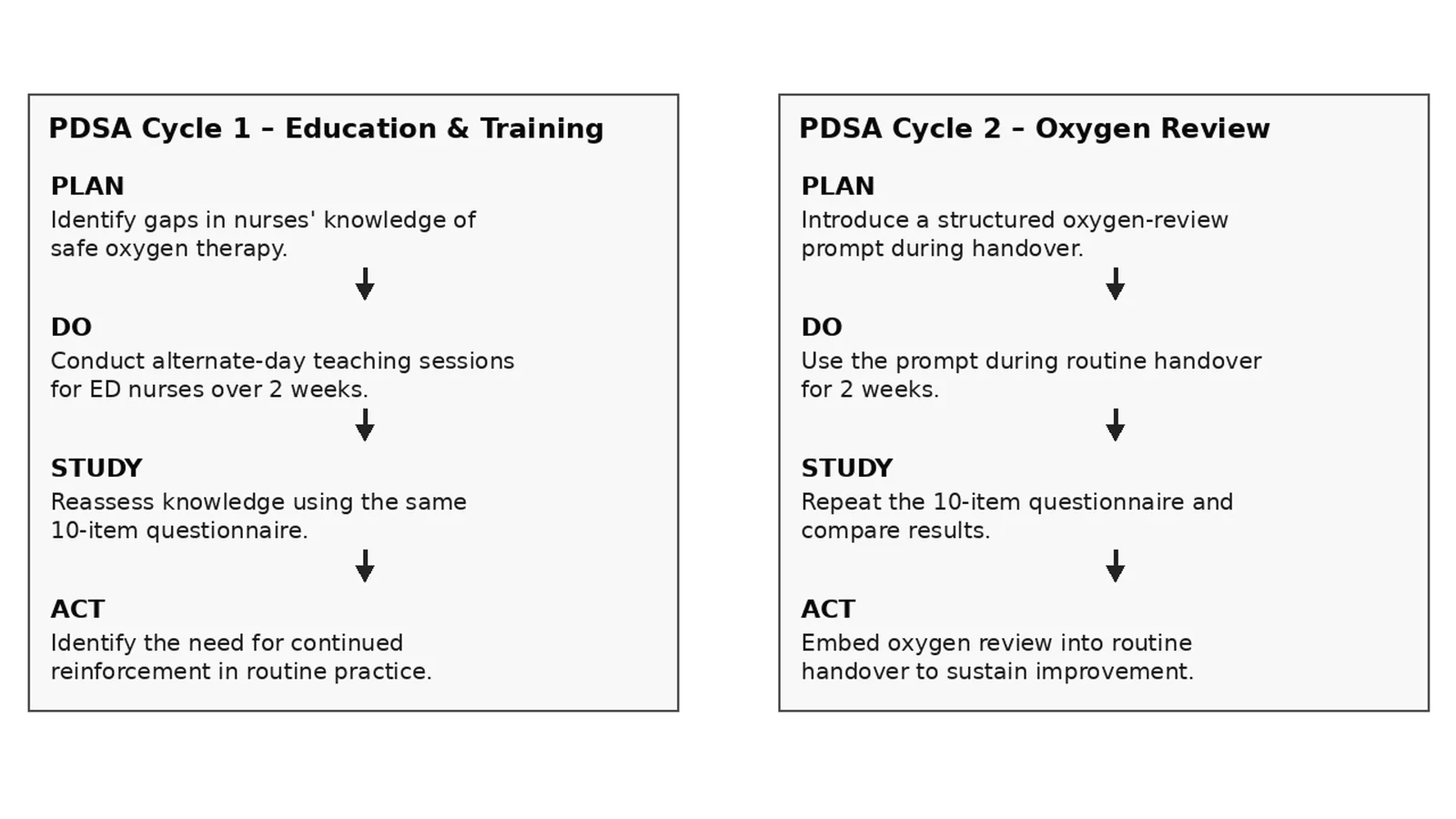
PDSA cycles for improving oxygen therapy practice Cycle 1 comprised education and training, while cycle 2 introduced a structured oxygen review prompt during handover. PDSA, Plan-Do-Study-Act; ED, emergency department

Data collection

Data were collected using the structured questionnaire administered at baseline, following PDSA cycle 1, and following PDSA cycle 2 to the same 30 participating nurses. Frequencies and percentages of nurses achieving satisfactory questionnaire performance (10/10) were calculated at each assessment stage.

The analysis was descriptive in nature and was intended to evaluate changes in overall questionnaire-based knowledge and awareness associated with the quality improvement intervention. No inferential statistical testing was performed.

The survey questionnaire and educational presentation have been included in the appendices as accessible Google Drive links for reference and reproducibility.

Ethical consideration

The project was reviewed by the Institutional Ethics Committee, which determined that it constituted a quality improvement/clinical audit initiative intended to improve health care delivery within the institution and did not involve human subjects research requiring formal Institutional Ethics Committee approval under institutional policy. Participation in the questionnaire assessment was voluntary, and informed consent was obtained from all participating nurses. No patient-identifiable information was collected, recorded, or analyzed as part of the project.

## Results

A total of 30 ED nurses completed the baseline questionnaire assessing their knowledge and practice regarding oxygen therapy.

At baseline, 18 of 30 nurses (60%) achieved satisfactory performance, defined as answering all 10 questionnaire items correctly. The baseline assessment identified areas requiring further education regarding safe oxygen therapy, including target oxygen saturation ranges, monitoring, potential harms of excessive oxygen administration, and oxygen reassessment and weaning.

Following the first PDSA cycle, which consisted of alternate-day structured teaching sessions conducted over two weeks, the proportion of nurses achieving satisfactory questionnaire performance increased from 18/30 (60%) at baseline to 22/30 (73.3%).

Following the second PDSA cycle, which incorporated a structured oxygen review prompt into routine clinical handover for a further two weeks, the proportion of nurses achieving satisfactory questionnaire performance increased further to 28/30 (93.3%).

Overall, questionnaire-based satisfactory performance increased from 60% at baseline to 73.3% following PDSA cycle 1 and 93.3% following PDSA cycle 2, as shown in Table 1. These findings indicate an improvement in questionnaire-based knowledge and awareness of safe oxygen therapy practices over the three assessment stages. However, actual bedside oxygen prescribing, reassessment, and weaning practices were not directly observed or audited.

**Table 1:** Overall questionnaire performance following the PDSA cycles (n=30) PDSA, Plan-Do-Study-Act

Assessment stage	Nurses answering all questionnaire questions correctly
Baseline	18/30 (60%)
Following PDSA cycle 1	22/30 (73.3%)
Following PDSA cycle 2	28/30 (93.3%)

## Discussion

This quality improvement project identified important gaps in nurses' knowledge and awareness regarding oxygen therapy in the ED. Although baseline awareness that oxygen should be treated as a medication and that oxygen saturation should be monitored was high, knowledge of appropriate target saturation ranges was less consistent, particularly for patients at risk of hypercapnic respiratory failure.

These findings are clinically relevant because inappropriate oxygen administration may expose patients to unnecessary treatment and potential harm. Evidence from a large systematic review and meta-analysis of randomized controlled trials found that liberal oxygen therapy was associated with increased mortality compared with a conservative oxygen strategy in acutely ill adults [3]. Hyperoxia has also been associated with adverse physiological effects and potentially poorer outcomes across several acute illnesses [4].

This supports the principle that oxygen should be titrated to an appropriate target rather than continued routinely once initiated. The British Thoracic Society (BTS) guideline recommends a target oxygen saturation of 94-98% for most acutely ill adults who are not at risk of hypercapnic respiratory failure, while a target of 88-92% is recommended for patients at risk of hypercapnic respiratory failure, pending further assessment and blood gas results where appropriate. National Institute for Health and Care Excellence (NICE) guidance similarly recommends maintaining oxygen saturation between 88% and 92% in patients receiving emergency oxygen during an acute exacerbation of COPD. These recommendations emphasize that oxygen should be titrated to a defined target range rather than administered liberally or continued without reassessment [5].

An important consideration highlighted by this project is the indication for initiating oxygen therapy. Oxygen should primarily be used to treat or prevent clinically significant hypoxemia rather than being administered routinely for symptoms such as breathlessness alone. The BTS guideline specifically notes that most non-hypoxemic patients who are breathless do not benefit from supplemental oxygen. Similarly, NICE guidance for acute chest pain advises against routine oxygen administration and recommends supplemental oxygen when oxygen saturation is below the appropriate threshold [6]. Therefore, the decision to administer oxygen should consider the patient's oxygen saturation, clinical condition, underlying disease, and risk of hypercapnic respiratory failure. Breathlessness should prompt assessment of the underlying cause rather than automatically resulting in oxygen administration when oxygenation is already adequate.

This distinction is particularly relevant in the ED, where patients frequently present with breathlessness, tachypnea, or anxiety and may initially receive oxygen before a complete assessment is available. While oxygen may be appropriate during the initial management of a genuinely hypoxemic or critically ill patient, it should subsequently be reduced to the lowest concentration required to maintain the appropriate target saturation once the patient has stabilized. The BTS guideline recommends reassessment when an increased concentration of oxygen is required and emphasizes the importance of adjusting oxygen therapy according to the patient's clinical response. Thus, oxygen therapy should be regarded as a dynamic treatment rather than a one-time intervention [7,8].

The baseline findings highlight an important educational need regarding patient-specific oxygen targets, particularly in patients with COPD who are at risk of hypercapnic respiratory failure and require controlled oxygen therapy within the recommended 88-92% saturation range [9]. Nurses therefore require an understanding not only of the target saturation range but also of why targets differ between patient groups and when oxygen should or should not be initiated.

The potential consequences of oxygen overuse also warrant consideration. Although oxygen is essential for correcting hypoxemia, increasing oxygen concentration beyond the level required to achieve the patient's target saturation does not necessarily provide additional clinical benefit. Excessive oxygen administration can result in hyperoxemia and may have physiological consequences, including pulmonary and systemic oxidative stress, vasoconstriction, and impaired tissue perfusion [10]. The BTS guideline therefore recommends avoiding unnecessarily high oxygen concentrations and maintaining patients within an appropriate target range rather than attempting to achieve the highest possible oxygen saturation.

The risk associated with excessive oxygen is particularly important in patients susceptible to hypercapnic respiratory failure. In COPD and other conditions associated with impaired ventilatory reserve, uncontrolled oxygen administration can worsen carbon dioxide retention through mechanisms including worsening ventilation-perfusion mismatch and the Haldane effect [11]. NICE notes that uncontrolled oxygen therapy during acute COPD exacerbations can reduce the depth and frequency of breathing in some patients, resulting in a rise in blood carbon dioxide and a fall in blood pH. NICE therefore recommends controlled oxygen therapy using an appropriate delivery device and flow rate to maintain a saturation of 88-92% during acute COPD exacerbations [12].

Importantly, oxygen overuse should not be viewed only in terms of COPD. Hyperoxemia may also be undesirable in other acute illnesses, and the BTS guideline specifically cautions against high concentrations of oxygen when they are not required to maintain adequate oxygenation. For example, in acute stroke, high concentrations should be avoided unless necessary to maintain normal oxygen saturation. This reinforces the broader principle that the goal of oxygen therapy is adequate oxygenation rather than maximal oxygenation.

Following the first PDSA cycle, which focused on targeted education and training, satisfactory performance on the 10-item oxygen therapy questionnaire increased from 60% (18/30) at baseline to 73.3% (22/30). The 13.3-percentage-point improvement suggests that targeted education was associated with improved questionnaire-based knowledge and awareness of safe oxygen therapy principles [13]. The teaching focused not only on oxygen saturation targets but also on practical reassessment, including consideration of the patient's current saturation, prescribed target range, oxygen delivery device, flow rate, clinical indication, and whether oxygen reduction or discontinuation was appropriate [14].

The second PDSA cycle focused on system-based reinforcement through incorporation of a structured oxygen review prompt into routine nursing and medical handovers. Following implementation, satisfactory performance on the 10-item oxygen therapy questionnaire increased further from 73.3% (22/30) after PDSA cycle 1 to 93.3% (28/30).

The further improvement observed following the second cycle suggests that reinforcing educational messages through an existing clinical process may have supported improved questionnaire-based knowledge and awareness. Rather than creating an additional standalone task, the intervention incorporated oxygen review into an established communication point. This may have reduced reliance on individual memory and created a shared expectation that oxygen requirements should be reviewed during handover. The finding is consistent with the BTS principle that target oxygen saturation should be prescribed and documented so that oxygen can be appropriately titrated during ongoing care.

The findings support the concept of oxygen stewardship, which involves assessing the indication for oxygen, selecting an appropriate target saturation and delivery device, monitoring the patient's response, and reducing or discontinuing oxygen when it is no longer required. As oxygen requirements may change with clinical improvement, regular reassessment is essential to minimize unnecessary oxygen exposure. Venturi masks are particularly useful when controlled oxygen delivery is required, including in patients at risk of hypercapnic respiratory failure, while reservoir masks may be required in severe hypoxemia or critical illness.

Overall, this project demonstrated that a combination of targeted education and a simple workflow-based prompt was associated with improved questionnaire-based knowledge and awareness of safe oxygen therapy, increasing from 60% at baseline to 93.3% following two PDSA cycles. The findings suggest that embedding oxygen reassessment into routine clinical handover may provide a feasible and practical strategy for promoting safer and more consistent oxygen therapy in a high-turnover ED environment. More broadly, the project reinforces that safe oxygen therapy requires more than recognizing hypoxemia: clinicians must consider whether oxygen is indicated, define an appropriate target, select the correct delivery method, monitor the patient's response, and actively reduce or discontinue oxygen when it is no longer required. This approach may help minimize both undertreatment of hypoxemia and unnecessary exposure to hyperoxemia, supporting more individualized and evidence-based oxygen therapy.

Limitation

There are, however, several limitations to this project. It was conducted in a single ED with a relatively small sample of 30 nurses, which may limit the generalizability of the findings. The questionnaire was developed for the project and was not formally validated or pilot tested. A possible Hawthorne effect may also have occurred, as participants were aware that their knowledge and oxygen-related practices were being assessed. In addition, the absence of a control group means that the observed improvement cannot be attributed solely to the interventions. The relatively short follow-up period also limited assessment of the long-term sustainability of the improvement and its impact on patient outcomes.

## Conclusions

This quality improvement project identified important gaps in oxygen therapy knowledge and the consistency of oxygen review and weaning among ED nursing staff. Targeted education was associated with an improvement in satisfactory questionnaire performance from 60% at baseline to 73.3% following the first PDSA cycle, with a further increase to 93.3% following the introduction of a structured oxygen review prompt during handover. These findings suggest that combining focused education with simple, workflow-based reinforcement can support improved knowledge and awareness of oxygen reassessment and weaning principles. Sustaining these interventions through periodic teaching, continued use of the handover prompt, and regular monitoring may further strengthen oxygen stewardship and promote safer, more appropriate oxygen use in the ED.

## Appendices

Survey link 

The questionnaire evaluated awareness of oxygen as a medication, appropriate target oxygen saturation ranges, potential harms of excessive oxygen administration, frequency of oxygen reassessment, and appropriate oxygen weaning practices. The same core questionnaire was used for the baseline and post-intervention assessments to facilitate comparison of changes following the Plan-Do-Study-Act (PDSA) cycles.

https://drive.google.com/drive/folders/1c8ax2t_S4AuvGrz82_-KForUhilkqfyp?usp=sharing

Teaching presentation link

The presentation focused on the principles of safe and appropriate oxygen therapy, including recognition of oxygen as a medication, prescribing and titrating oxygen to a target oxygen saturation range, appropriate target saturations for different clinical conditions, selection and use of oxygen delivery devices, and the risks associated with excessive oxygen administration. Particular emphasis was placed on controlled oxygen therapy in patients at risk of hypercapnic respiratory failure, appropriate oxygen monitoring and reassessment, and timely reduction or discontinuation of oxygen when clinically indicated. The presentation also included practical guidance on oxygen delivery devices and an approach to persistent hypoxemia despite supplemental oxygen.

https://drive.google.com/drive/folders/1TLNK408shK3gsypMnqFWjm9rPlbuiXmI?usp=sharing
